# Neuraminidase Inhibitor Resistance in Influenza: Assessing the Danger of Its Generation and Spread

**DOI:** 10.1371/journal.pcbi.0030240

**Published:** 2007-12-07

**Authors:** Andreas Handel, Ira M Longini, Rustom Antia

**Affiliations:** 1 Department of Biology, Emory University, Atlanta, Georgia, United States of America; 2 Program of Biostatistics and Biomathematics, Fred Hutchinson Cancer Research Center, Seattle, Washington, United States of America; 3 Department of Biostatistics and School of Public Health and Community Medicine, University of Washington, Seattle, Washington, United States of America; ETH Zürich, Switzerland

## Abstract

Neuraminidase Inhibitors (NI) are currently the most effective drugs against influenza. Recent cases of NI resistance are a cause for concern. To assess the danger of NI resistance, a number of studies have reported the fraction of treated patients from which resistant strains could be isolated. Unfortunately, those results strongly depend on the details of the experimental protocol. Additionally, knowing the fraction of patients harboring resistance is not too useful by itself. Instead, we want to know how likely it is that an infected patient can generate a resistant infection in a secondary host, and how likely it is that the resistant strain subsequently spreads. While estimates for these parameters can often be obtained from epidemiological data, such data is lacking for NI resistance in influenza. Here, we use an approach that does not rely on epidemiological data. Instead, we combine data from influenza infections of human volunteers with a mathematical framework that allows estimation of the parameters that govern the initial generation and subsequent spread of resistance. We show how these parameters are influenced by changes in drug efficacy, timing of treatment, fitness of the resistant strain, and details of virus and immune system dynamics. Our study provides estimates for parameters that can be directly used in mathematical and computational models to study how NI usage might lead to the emergence and spread of resistance in the population. We find that the initial generation of resistant cases is most likely lower than the fraction of resistant cases reported. However, we also show that the results depend strongly on the details of the within-host dynamics of influenza infections, and most importantly, the role the immune system plays. Better knowledge of the quantitative dynamics of the immune response during influenza infections will be crucial to further improve the results.

## Author Summary

Neuraminidase Inhibitors (NI) are currently the most effective drugs against influenza. Recent cases of NI resistance are a cause for concern. A number of studies have reported the fraction of treated patients from which resistant virus could be isolated. While these results provide some assessment of the danger of NI resistance, a more quantitative understanding is preferable. We specifically want to know how likely it is that an infected, treated patient infects another person with the resistant strain, and how likely it is that the resistant strain subsequently spreads. Knowing these quantities is important for studies of the population-wide emergence of resistance. While these parameters can often be estimated from epidemiological data, such data is lacking for NI resistance in influenza. Here, we use an alternative approach that combines data from influenza infections of human volunteers with a mathematical framework. We find that the initial generation of resistant cases is most likely lower than the fraction of resistant cases reported. However, our study also clearly shows that the results depend strongly on the role the immune response plays, an issue that needs to be addressed in future studies.

## Introduction

Neuraminidase Inhibitors (NI) are currently the most effective drugs against influenza [1]. They also constitute an important component of control strategies against a potential pandemic [2]. However, cases of NI resistance have already been reported, for both currently circulating human influenza [3] and the avian H5N1 strain [4]. Mathematical models and computer simulations have been used to study how NI treatment or prophylaxis might affect the spread of resistance in a population [5–8]. The accuracy of the predictions obtained from these studies depends on the accuracy of the estimates for the parameters governing the model dynamics.

Two important parameters, for which there are currently no good estimates, are: (i) the initial generation of resistance, defined here as the number of resistant infections caused by a patient receiving NI treatment who was initially infected with a sensitive strain; (ii) the subsequent spread of resistance, defined as the number of resistant infections caused by a patient initially infected with the resistant strain.

The best data we currently have for the generation of NI resistance come from clinical studies that report the fraction of treated patients from which resistant strains could be isolated [9–13]. Unfortunately, the results strongly depend on the details of the study, such as sensitivity of virus detection [3]. Further, knowing the fraction of patients that harbor resistant virus does not directly lead to an estimate for the generation of resistance.

For the spread of NI resistant strains, some insights have been obtained from studies with ferrets, where it was shown that certain resistant strains are transmissible, while others are not [14–16]. However, these studies currently do not provide enough quantitative data to allow estimation of the parameter governing the spread of resistance.

The dilemma is obvious: We need good parameter estimates to understand and model the potential spread of NI resistant influenza, but we do not want to wait until such spread has occurred and epidemiological data are available that would allow us to obtain good parameter estimates. Therefore, it is important to find alternative ways to estimate these parameters.

Here, we use a conceptual framework that links within-host infection dynamics to between-host epidemiological parameters [17–20]. We extend this framework and combine data from influenza infection of human volunteers with mathematical models. We show how this approach can produce estimates for the generation and spread of resistance, without the need for epidemiological data. Our study also shows that a better understanding of the within-host infection dynamics of influenza is crucial if we want to obtain precise results using this approach.

## Materials and Methods

### Deterministic Model of Infection Dynamics

The simplest way of modeling the dynamics of viral infections is based on ordinary differential equations, an approach that has a long and successful history [21,22]. The basic model describes the dynamics of uninfected and infected cells, and virus. In this model, virions infect target cells (in the case of influenza, these are mainly epithelial cells of the respiratory tract), leading to depletion of those cells and subsequent decline in viral load. We use a version of this simple model, which we refer to as the target cell depletion (TD) model. A recent study showed that such a simple model could fit influenza viral load data [23].

This TD model does not include an immune response. Since the immune response likely contributes to viral clearance [24,25], we also use a second model that includes an antibody-based immune response. We assume the antibody levels increase in the simplest way possible, through growth at a constant rate, independent of viral load (similar to the “programmed response” expansion found in T cells [26]). This model, which we term the immune response (IR) model, has more parameters than the TD model. The data we use to fit the models is not sufficient to allow discrimination between the two models; therefore, the IR model cannot be justified on statistical grounds. However, since a large number of studies have indicated the importance of immune responses for viral clearance, we find it important to also study this model. The equations and parameters for the two models are given in Tables 1 and 2. For general information on models of this type, and their use to describe viral infection dynamics, we refer the reader to the existing literature [21,22].

**Table 1:**
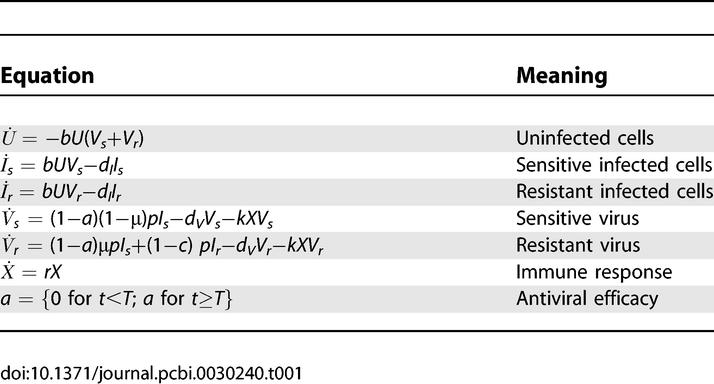
Equations for the Two Models Describing the within-Host Dynamics

**Table 2:**
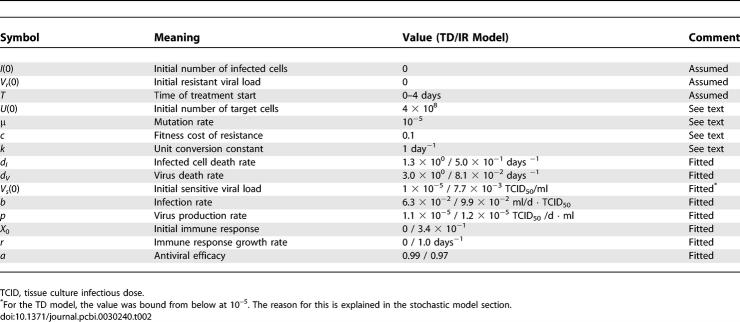
Parameters for the Two Models Describing the within-Host Dynamics

Most parameter values for the two models are obtained by fitting viral load data from human volunteers [27]. We include datapoints from the experimental study for which the 95% confidence interval is above the level of assay detection for a single individual (see [27] for details). The data are shown as symbols in Figure 1, together with the best fit viral load curves. The parameter values for the best fits are given in Table 2.

**Figure 1:**
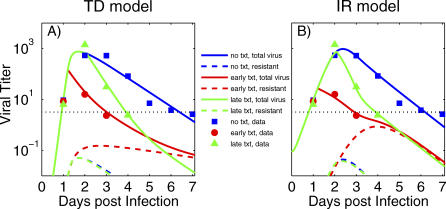
Viral Titer (TCID_50_/ml of Nasal Wash) Symbols in (A) and (B) are data from humans infected with influenza A/Texas/91 (H1N1). Early drug treatment starts at around 29 h, late treatment at around 50 h post-infection. The dotted horizontal line shows the limit of assay sensitivity. The solid lines show total viral load as obtained from (A) the target-cell depletion (TD) model and (B) the immune response (IR) model. The dashed lines show the resistant subpopulation. Further details on the experimental data are given in [27].

While we tried to obtain all parameters through fitting, the available data are not sufficient to obtain reliable estimates. We therefore decided to use estimates obtained from the literature for those parameters where such information was available. One parameter that we estimate from independent experimental studies is the mutation rate, μ, at which NI resistant mutants are produced. The mutation rate per base pair per replication for influenza A has been estimated to be about 7 × 10^−5^ [28,29]. A more recent study reported the rate to be about 2 × 10^−6^ [30]. These studies come with considerable uncertainty due to only a few observed mutation events. Several mutations conferring neuraminidase resistance have been identified [3,31]. Still, the number of possible mutations that results in NI resistant, viable mutants is likely small. If we estimate that number to be between 1 and 10, we obtain a mutation rate in the range of about 10^−6^ to 10^−4^. For most of our simulations, we choose *μ* = 10^−5^. We also investigate how variation in μ affects the results.

Another independently estimated parameter is the cost in fitness, *c*. We assume that the dynamics of resistant virus is the same as that of the drug-sensitive one, with the exception that the virus production rate is lowered by 1 − *c*. The parameter *c* represents the fitness cost that comes with being resistant. (Similar results are obtained if we assume that resistance reduces fitness by lowering the infection rate *b.*) Several studies have indicated that some NI resistant mutants have a significantly reduced fitness, while others have fitness similar to the sensitive strain [14,16,31–33]. From recent in vivo studies, one can obtain estimates for the fitness cost. Fitting the IR model to viral load data from ferrets that were infected with either wild-type influenza or resistant mutants (see Figure 5 in [16]), we obtained a fitness cost of 49% for the R292K mutant and a 9% fitness cost for the E119V mutant (see [16] for details on these mutants). To obtain conservative results for our study, we chose the fitness cost for the resistant strain to be 10% (*c* = 0.1). We also study how different values of *c* influence the results.

The initial number of uninfected epithelial cells has been estimated previously to be *U*_0_ = 4 × 10^8^ [23]. Performing an independent estimate that assumes a surface area of the upper respiratory tract of 100–200 cm^2^ [34,35] and the size of an epithelial cell of 1 − 5 × 10^−7^ cm^2^ [36], we obtain values in the same range. For sake of consistency with the earlier study, we choose *U*_0_ = 4 × 10^8^. While this estimate comes with a certain amount of uncertainty, for our purposes, knowing the exact value for *U*_0_ is not critical since a different value would simply lead to a rescaling of some of the parameter values.

Lastly, the constant *k* is set to *k* = 1 per day. It is only used to make units consistent. Any change in *k* would only lead to a rescaling of the immune response, which is given in arbitrary units.

### Stochastic Model of Infection Dynamics

Since resistant virions are initially not present and, upon initial generation, are at low numbers, stochastic effects can become important. It is therefore useful to also use stochastic versions of the deterministic models described in the previous section. One problem with using stochastic simulations is the issue of units. With the deterministic models introduced above, we are able to work in the experimentally reported units of 50% tissue culture infectious doses per milliliter (TCID_50_/ml) of nasal wash. However, if we want to study the impact of stochastic effects, we need to convert to numbers of infectious virions at the site of infection. Both TCID_50_ measurements as well as our models only deal with viable, infectious virions. Non-infectious viral particles, which are known to be created in rather large quantities due to the segmented nature of the influenza virus, can therefore be ignored. Still, it is unclear how TCID_50_/ml of nasal wash convert to numbers of infectious virions. At the minimum, one TCID corresponds to a single infectious virion, but it is more likely that on average more than one virion is needed to establish an infected cell culture. We estimate that 1−100 virions correspond to one TCID. Next, virions/ml of nasal wash need to be converted to virions/ml at the site of infection, which for uncomplicated influenza infections is mainly the upper respiratory tract. Not much information is available; based on circumstantial data ([37,38]), we estimate that the concentration at the site of infection is higher by a factor of 1−100. Finally, the volume of the upper respiratory tract has been estimated to be about 30 ml [35]. Combining it all, we obtain the very rough estimate that 1 TCID_50_/ml of nasal wash corresponds to about 10^2^–10^5^ virions at the site of infection. Calling this conversion factor γ (with units of (TCID_50_/ml)^−1^), the variables of the deterministic model are rescaled for the stochastic model according to V → *γV*, *p* → *γp*, *b* → *b/γ*. A smaller γ leads to fewer virions in the stochastic simulation, thereby increasing the importance of stochastic effects.

An important issue is the fact that even for the largest estimate of γ, the best fit value for the initial viral load in the TD model (*V*_0_ *=* 4.9 × 10^−7^ TCID_50_/ml) corresponds to an inoculum size of less than one virion. While some studies suggest that a few virions are enough to start an infection, clearly a value below one makes no sense. It is likely that the initial estimate for *V*_0_ obtained from the model is wrong. Indeed, experimental data from mice suggests that instead of having minimum viral load at the start of infection, the viral load first drops over the course of a few hours, before it starts to increase again [39]. Since the earliest data point available is at 24 h post-infection, we cannot resolve such dynamics, therefore leading to a likely underestimate for *V*_0_. Another reason suggesting that the value for *V*_0_ obtained from the TD model is too low comes from the fact that for the IR model, the value of *V*_0_ is orders of magnitude larger. While this indicates problems with the TD model, the data used here do not allow us to conclusively reject it. We return to the problem of model discrimination and lack of data in the Discussion. To allow comparison between the deterministic and stochastic versions of the TD model, we bound *V*_0_ by 10^−5^ TCID_50_/ml from below. This leads to a fit that is only a few percent worse than the unbounded fit, and by setting *γ* = 10^5^, we can then compare deterministic and stochastic results in the TD model. Further, to investigate the impact of a lower γ, we set *γ* = 10^3^ for the IR model.

### Numerical Implementation of the Models

The deterministic models are fitted to the viral load data using several fitting routines (lsqnonlin, fmincon, and nlinfit) provided by Matlab R2006b (The Mathworks). To obtain the results shown below, we perform both stochastic and deterministic simulations. The deterministic ODEs are implemented in Matlab R2006b, the stochastic simulations are written in Fortran 90. A purely stochastic simulation (Gillespie algorithm) would be prohibitively slow, due to the large numbers of cells/virions. Therefore, the simulation is implemented using a partitioned leaping algorithm [40]. The results shown for the stochastic simulations are averages over 1,000 or 5,000 runs. Since our focus is on stochastic effects of the virus dynamics during resistance emergence, we treat the immune dynamics in the IR model deterministically. All programs are available from the authors upon request.

## Results

### Modeling Virus Shedding

The models described in the Materials and Methods section provide us with viral load data during the course of the infection. We can then relate viral load to the amount of viral shedding. The amount of shedding depends on host symptoms, such as sneezing or coughing. These symptoms are caused by host response mechanisms, which in turn depend on viral load [41]. To find the relation between viral load and shedding, we can use data from two recent studies that report viral load as well as nasal discharge weight for volunteers infected with influenza A/Texas/36/91 (H1N1) [42,43]. By plotting nasal discharge as a function of viral load, we can fit a function to obtain an analytic relationship. For low levels of virus, we expect few symptoms to occur, resulting in low viral shedding. Once viral load reaches a certain level, symptoms start to appear and shedding increases. For high viral load, shedding will likely saturate. We can model this using a sigmoid function, such as a four-parameter Hill function given by

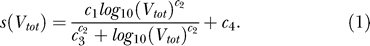



Here, *V_tot_* is the total viral load (both sensitive and resistant virus). The best fit values for the four parameters are found to be *c*_1_ = 6.1, *c*_2_ = 4.8, *c*_3_ = 2.6, and *c*_4_ = 1.5. The data and best fit are shown in Figure 2. We also fitted a linear function to the data; however, the function given by Equation 1 produces a statistically slightly better fit (adjusted *R* ^2^ is 0.75 for the Hill function and 0.73 for a linear model). We also prefer Equation 1 on biological grounds, due to its threshold and saturation effects for low and high viral load, respectively.

**Figure 2:**
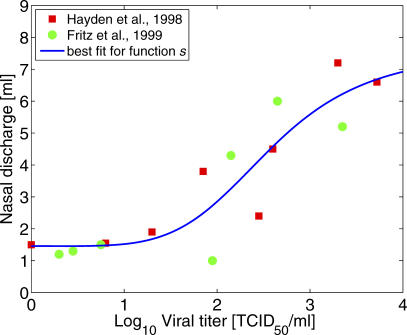
Nasal Discharge Weight as a Function of Viral Load Data are from [42] (squares) and [43] (circles). Also shown is the best fit for the function *s* (Equation 1). Note that daily discharge is measured in grams; we make the approximation that one gram corresponds to 1 ml in volume.

We then obtain the total amount of viral shedding by multiplying virus concentration with the amount of discharge at every time point and integrating over the duration of infection. The equation for the total amount of shedding is given by

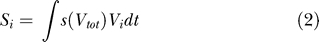
where *i* = *r* for the resistant and *i* = *s* for the sensitive strain. It is important to note that the amount of either sensitive or resistant shedding depends on the *total* viral load of both sensitive and resistant virus. In the following, we will consider shedding of sensitive virus in the absence of treatment (*S_s_*), shedding of resistant virus during a treated infection started by sensitive virus (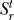 ), and shedding of resistant virus during an infection started by resistant virus (*S_r_*).

### Connecting Shedding with Epidemiological Parameters

The results obtained for viral shedding allow us to estimate the two quantities of interest, the initial generation of resistance and its subsequent spread through the population. Both quantities can be expressed in terms of the average number of new infections caused by an infected host, the reproductive number, *R* [44]. The simplest relation between *R* and shedding, *S*, is a direct proportionality, *R* = *kS*. (In Appendix A: Mapping Behavior to Viral Load, we discuss a more detailed relation including a behavioral component.) The parameter *k* describes the rate of transmission for the sensitive (*k_s_*) or resistant (*k_r_*) strain. For the sensitive strain in the absence of treatment and resistance, we have *R_s_* = *k_s_S_s_*. The generation of resistance, 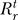 , can be expressed as the expected number of resistant infections caused by transmission of resistant virus (characterized by the transmission rate *k_r_*) from a treated patient initially infected with sensitive virus, who sheds resitant virus, 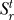 . This leads to 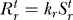 . The subsequent spread of resistance is expressed as the expected number of resistant infections caused by a host initially infected with only the resistant virus, *R_r_* = *k_r_S_r_*. Figure 3 shows these different processes schematically.

**Figure 3:**
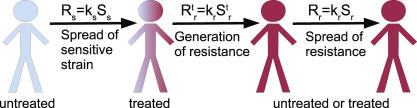
Schematic of Resistance Generation and Spread

Estimates for the reproductive number of influenza (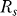 ) are available and our framework allows us to determine the amount of shedding, *S_s_*. This allows calculation of the transmission rate, *k_s_*. It is not clear how the ability to spread as measured by *k_s_* or *k_r_* differs between sensitive and resistant strains. Studies in ferrets have shown that transmissibility of resistant strains varies, some resistant mutants are poorly transmitted, while others seem to be as well transmitted as the sensitive strain [14–16]. As a (likely very conservative) bound for the potential of transmission between humans, we assume in what follows that the resistant strain transmits as well as the sensitive strain, i.e., *k_r_* = *k_s_*. One can easily change this assumption, the results scale accordingly. With this assumption, we can then write 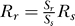 and 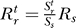 , allowing us to determine the generation of resistance during treatment as measured by 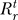 and its subsequent spread as measured by *R_r_*.

### The Generation of Resistance

We first consider the generation of resistance during treatment as described by 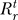 . To that end, we determine shedding of sensitive virus in the absence of both antiviral drug and resistant virus, *S_s_*, as well as shedding in the presence of treatment and resistant virus, 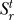 . Figure 4 shows the generation of resistance as a function of treatment start, antiviral efficacy, fitness cost, and mutation rate for the TD and IR models for both deterministic and stochastic simulations. For the shown results, we use a value of *R_s_* = 2, in line with current estimates [45,46].

**Figure 4:**
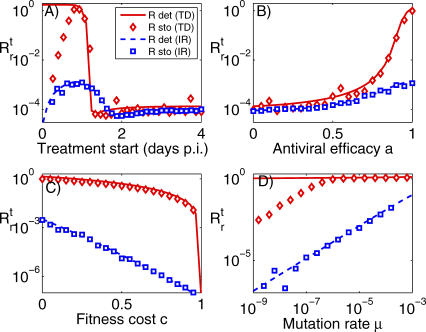
Generation of Resistance, as Measured by 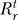, for the TD Model (No Immune Response) and IR Model (with Immune Response) Results are shown for both deterministic and stochastic simulations. Unless varied, treatment starts 24 h post-infection, and the other parameters are as shown in Table 2.

Several observations are notable. First, the results show that more effective treatment, which better removes the sensitive strain and thereby allows the resistant strain to grow, increases the danger of resistance generation (Figure 4B). A similar finding holds for the timing of treatment start (Figure 4A). Treatment that starts late has little impact on the sensitive population, which prevents the resistant population from reaching high numbers. Treatment that starts less than two days after infection has an impact, and initially increases the generation of resistance. Interestingly, the results obtained for the IR model and the stochastic TD model suggest that very early treatment can reduce the danger of resistance generation. For the IR model, this is because if treatment reduces the sensitive strain fast enough, the immune response is able to quickly eradicate the remaining resistant subpopulation. For the TD model, early treatment can lead to eradication of the sensitive strain before any resistant mutants are created, resulting in significantly lower levels of resistance generation compared to the deterministic model, where resistant mutants are always created.

Second, a change in fitness cost or mutation rate has little impact on the TD model for a wide range of parameter values but does affect the results for the IR model (Figure 4C and 4D). For the TD model, the stochastic results deviate from the deterministic model for low mutation rates, again because resistant mutants are often not created.

Third, the TD model consistently predicts values for resistance generation above those for the IR model. This can be understood as follows: in the TD model, the resistant strain competes with the sensitive strain for resources (target cells). In the presence of the sensitive strain, the resistant strain is outcompeted. If the sensitive strain is removed, the resistant strain will infect most target cells and reaches high levels. In contrast, the immune response in the IR model acts against both the sensitive and resistant strains. If sensitive virus is suppressed by NI, the mounting immune response will still act against the resistant strain, preventing it from reaching high levels [47].

Fourth, stochastic effects become important either if treatment occurs early and wipes out the sensitive population before resistance has been created, or if mutation rates are low. As mentioned in the Materials and Methods section, the conversion factor from TCID to virions can only be estimated rather broadly. If this factor is set to a lower value, the importance of stochastic effects increases further (unpublished data).

### The Spread of Resistance

While the parameter governing the generation of resistance is important, the parameter describing the subsequent spread of resistance is arguably more important. Even if generation of resistance is infrequent, only a few resistant infecteds could be enough to start a resistant outbreak. The possibility for such an outbreak is determined by *R_r_*, the number of secondary infections caused by a person infected with resistant virus. To determine *R_r_*, we performed simulations assuming that the host is infected with only the resistant strain. This allowed us to obtain *S_r_*, and using the previously obtained values for *S_s_*, we can then compute *R_r_*. In Figure 5, we plot *R_r_* for the two models. If *R_r_* < 1, chains of transmission of resistant virus will stutter to extinction. The results show that this is the case if the fitness cost is at least ≈25% for the IR model or at least ≈45% for the TD model. While some NA resistant mutants have been found to carry a significant within-host fitness cost, other mutants are similar in within-host fitness to the wild-type strain [16,31], which suggests that these mutants might have the potential to spread—provided transmission of the sensitive and resistant strains are equal (*k_s_* = *k_r_*), something that seems to be the case for some but not all resistant mutants [14–16]. While a value of *R_r_* > 1 can lead to an outbreak in a fully susceptible population, during a pandemic the time at which resistance appears will be crucial. If a significant number of susceptible hosts have already been infected with the sensitive strain, the effective reproductive number might not be enough for a subsequent, resistant outbreak [7,48]. However, once a resistant strain has been created and spreads, it is possible that further mutations occur. While back-mutations to the fitter, susceptible strain are possible, there is evidence that it is often more likely that instead of reversion to the original form, the resistant mutant undergoes further, so called compensatory mutations [49,50]. These mutations reduce the fitness cost that comes with resistance, while at the same time retaining the resistant mutation. The result can be a strain that is at the same time resistant and has a fitness similar to the initial, sensitive strain. We have previously considered some of the implications of compensatory mutations on the spread of resistance through a population [51]. Limited in vitro evidence suggests that compensatory mutations might occur for NI resistant influenza [52].

**Figure 5:**
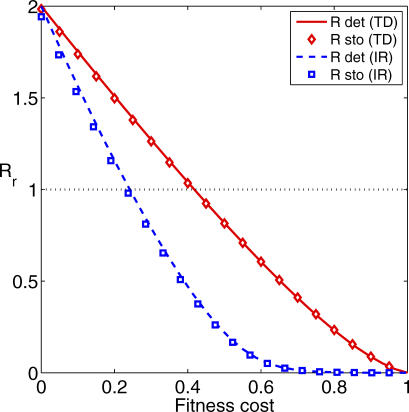
Spread of Resistance as Measured by *R_r_* Note that only variation in the fitness cost has an impact on *R_r_* since treatment does not affect the resistant strain.

## Discussion

We have demonstrated that it is possible to combine data from infected individuals with mathematical models to obtain estimates for important between-host parameters, without the need for epidemiological data.

The results we obtained suggest that to minimize the danger of resistance generation, treatment at the very beginning of the infection (i.e., prophylaxis) is best (Figure 4A). While this seems to reduce the changes of generating resistance, once it has been generated, it is likely to spread, as long as the ability of the resistant strain to transmit is similar to that of the sensitive strain (Figure 5).

Unfortunately, several shortcomings currently do not allow us to obtain precise results. The main problem is our lack of understanding of the dynamics governing within-host influenza infections. Both the TD model without immunity and the IR model with immunity are able to fit the data; however, the estimated parameters differ. Additionally, parameters such as the conversion rate between TCID_50_ and number of virions, or the rate of mutation, are based on estimates that come with a significant amount of uncertainty. The problem of unrealistic parameter estimates, such as the very low initial viral load obtained for the unbounded TD model, further reinforce the fact that more data is needed to better discriminate between models.

The inability to discriminate between models would not be too problematic if the two different models produced similar results. While the results are somewhat similar for the spread of resistance, as well as the impact of treatment on the sensitive strain (see Appendix B: The Impact of Treatment on the Spread of the Sensitive Strain), they differ significantly with regard to the initial generation of resistance (Figure 4). Inclusion of an immune response reduces the danger of resistance generation. Since there is no immunity in the TD model, the results obtained from this model provide an upper bound. We expect the “true” parameter values to be closer to those of the IR model. While we believe that a model with an included immune response is more accurate compared to the TD model, it is by no means clear that an antibody-based response is the most important component. Many studies point toward the fact that innate, cellular, and humoral immune responses all play important, potentially overlapping roles to help clear the infection. In future studies, it will be crucial to obtain more data for the within-host dynamics, to allow for better model discrimination and a better understanding of the important drivers of the infection dynamics. Such an improved understanding of the within-host dynamics of influenza will likely require a tight combination of experimental data with mathematical models [23,53].

Also needed are further studies that investigate the ability of the resistant strains to transmit. Specifically, it is necessary to understand if reduced transmission is due to reduced shedding, reduced survival of the resistant strain during transmission, or other factors such as changes in contact rates. To that end, further studies in ferrets seem to be the most promising approach.

While the lack of better data currently prevents us from obtaining quantitative results, these limitations can be overcome. Provided enough experimental data on within-host dynamics and some transmission data between individuals are available, the approach discussed here can produce parameter estimates that can then be used to simulate and study potential spread of novel emerging pathogens. Crucially, this can be done before the pathogen has produced outbreaks large enough to reliably obtain parameter estimates from epidemiological data. Such an approach will be important if we want to be one step ahead of NI resistant influenza, a potential H5N1 outbreak, and other newly emerging diseases for which epidemiological data are lacking. In the best case, we will be able to prevent this data from ever existing.

### Appendix A: Mapping Behavior to Viral Load

In the previous text, we connected viral shedding and the number of new infections by a simple proportionality, *R* = *kS*. Here we consider a more complicated mapping that includes behavioral changes. A sick person might reduce the frequency of contacts with other persons, for instance by staying at home instead of going to work. Such a self-imposed quarantine reduces the ability to infect other people. Since contact reduction is likely dependent on the strength of symptoms, we use symptom score as a proxy for behavior [42,43]. If the symptom score is zero, an infected person “feels fine” and behaves as usual. As symptoms increase, the contact rate is reduced. Unfortunately, there is no data that reports how changes in symptom scores influence contact rates. We therefore use the rather “ad hoc” relation *w* = 1 / (1 + *y*) between (normalized) contact rate, *w*, and symptom score, *y*. With the right data available, one could improve this relation by for instance introducing additional scaling constants, or by choosing an entirely different mapping from symptoms to behavior. Since such data is absent, the results obtained from this approach should only be considered illustrative.

We can express the symptom score *y* as a function of viral load *V* by fitting a function *y*(*V*) to data reported in [42,43]. While symptom score is unlikely to depend exponentially on viral load over a wide range, for the reported data we obtained a good fit for an exponential function given by *y* = *f*_1_ *exp*[*f*_2_ *log*_10_(*V*)] with best fit parameter values given by *f*_1_ = 0.15 and *f*_2_ = 0.77 (Figure 6). Also note that symptoms are unlikely directly related to viral load, but instead are more likely to be caused by depletion of target cells or the immune response. We use viral load here as a proxy for those (unknown) quantities, which allows us to use available data and present the results in a closed framework.

**Figure 6:**
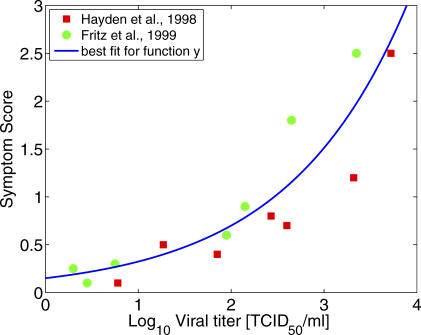
Symptom Score as a Function of Viral Load Data are system symptom score values and viral load from [42] (squares) and [43] (circles). Also shown is the best fit for the function *y*.

We then obtain *w* as a function of viral load as

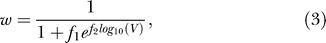
and the number of secondary infections is given by

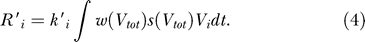



The integral expression now represents shedding, adjusted for behavioral changes. The constant *k*′*_i_* includes factors such as survival of the strain outside the host. If we again assume *k*′*_s_* = *k*′*_r_*, we have *R*′*_r_ = D*′*R_s_* and 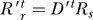 where *D*' is given by

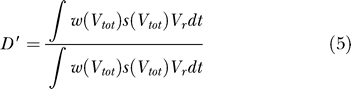
and an equivalent expression for *D*′*^t^*. Figure 7 compares results for 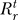 and 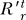 for the case of the IR model. One can see that including the behavioral component only changes results slightly. We found the same to be true for all the other results presented (unpublished data).

**Figure 7:**
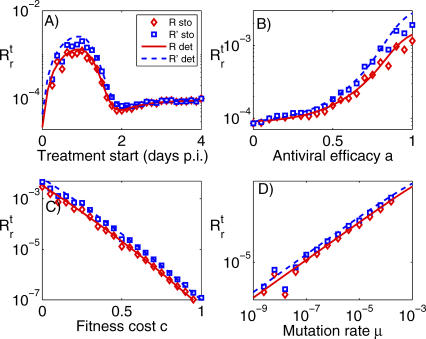
Generation of Resistance for the IR Model Shown are results for the model without (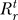) and with (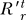) behavioral component for both the stochastic and the deterministic simulations. Unless varied, treatment starts 24 h post-infection, the fitness cost is *c* = 0.1, antiviral efficacy is *a* = 0.97, and the mutation rate is μ = 10^−5^.

### Appendix B: The Impact of Treatment on the Spread of the Sensitive Strain

While the main focus of this study is on the generation and spread of NI resistant influenza, the framework can also be used to study how treatment affects shedding and therefore transmission of the sensitive strain, providing an approach that is complementary to existing ones [54]. Ignoring the resistant strain for this calculation, we define 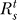 as the expected number of new infections created by an infected patient who receives treatment. Figure 8 shows 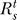 as a function of treatment start and antiviral efficacy. As expected, early treatment and high antiviral efficacy can significantly reduce transmission. However, even for the rather high antiviral efficacies of 99% and 97%, respectively, treatment within the first ≈48 h is required to reduce 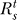 below one. To stop spread in a population, such early treatment would need to be applied to almost every infected person, something that is not feasible. This suggests that using treatment alone is unlikely to stop an outbreak. Rather, a combination of treatment, prophylaxis, vaccination, and social distancing measures will be required to effectively prevent the next pandemic, as has been noted previously [55,56]. One additional interesting result seen in Figure 8 is the fact that differences between the TD and IR models are less pronounced compared with the results obtained for resistance generation. This is because the presence or absence of immune pressure has a stronger effect on the potential de novo emergence of the resistant strain.

**Figure 8:**
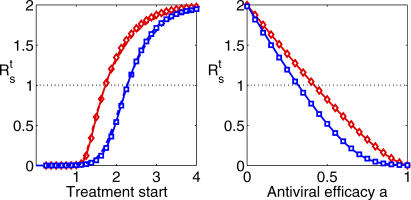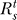 as a Function of Treatment Start and Antiviral Efficacy Unless varied, treatment starts 24 h post-infection, and antiviral efficacy is *a* = 0.99/0.97. Red = TD model, blue = IR model.

## Abbreviations

IR: immune response
NI: neuraminidase inhibitor
TCID: tissue culture infectious dose
TD: target cell depletion
